# Elevated plasma levels of lysophosphatidic acid and aberrant expression of lysophosphatidic acid receptors in adenomyosis

**DOI:** 10.1186/s12905-017-0474-z

**Published:** 2017-11-25

**Authors:** Bicheng Yang, Liqun Wang, Xiaoju Wan, Yunjun Li, Xiaohong Yu, Yunna Qin, Yong Luo, Feng Wang, Ouping Huang

**Affiliations:** Jiangxi Provincial Maternal and Child Health Hospital, No. 318 Bayi street, Nanchang, Jiangxi 330006 People’s Republic of China

**Keywords:** Lysophosphatidic acid, Adenomyosis, Endometrium, Plasma

## Abstract

**Background:**

Given the important roles of the receptor-mediated lysophosphatidic acid (LPA) signaling in both reproductive tract function and gynecological cancers, it will be informative to investigate the potential role of LPA in the development of adenomyosis. The objective of this study was to evaluate the levels of LPA in plasma and the expression of six LPA receptors in the endometrial tissue collected from women with and without adenomyosis.

**Methods:**

Plasma and endometrial tissue samples were collected form women with and without adenomyosis. The levels of LPA in plasma were determined by using high-performance liquid chromatography electrospray ionization tandem mass spectrometry (HPLC-ESI-MS/MS). Immunohistochemistry was performed to evaluate the expression of six LPA receptors (LPA1–6) in endometrial tissue samples. The effects of LPA on IL-8 production, VEGF production and cell proliferation in human endometrial stromal cells (ESCs) were also assessed.

**Results:**

LPA1 staining was localized to the cytoplasm, membrances of the epithelial cells of the endometrial glands, and there was little staining in the stromal cells. LPA2–5 staining were localized to the nuclei of stromal and glandular cells. Plasma levels of LPA were increased in adenomyosis. LPA1, LPA4 and LPA5 immunoreactivity were significantly higher in the adenomyosis group than in the control group, while LPA2 and LPA3 immunoreactivity were significantly lower in the adenomyosis group than in the control group. LPA6 was undetectable in the endometria. LPA induced the release of IL-8 from ESCs but did not affect cell proliferation and VEGF production.

**Conclusion:**

These results indicate that elevated plasma levels of LPA and aberrant expression of LPA receptors in the endometria may be associated with the development of adenomyosis.

## Background

Adenomyosis is a common benign condition in women of reproductive age and it characterized by the presence of endometrial glands and stroma within the uterine myometrium [1, 2].Adenomyosis is a serious disease for women because of dysmenorrheal, infertility, abnormal uterine bleeding, chronic pelvic pain and menorrhagia. A series of hypotheses that attempt to explain the genesis of adenomyosis has been established [3]. However, the etiology and pathogenesis of the disease remain poorly understood.

LPA is a simple phospholipid that exerts many physiological and pathological actions on various cell types, such as cell proliferation and differentiation [4], cytoskeletal rearrangement [5], cell-to-cell interaction [6], and tumorigenesis. So far LPA has been detected in many various biological fluids such as serum and plasma, tears, ascites, seminal plasma, and follicular fluid. Moreover, it can also be produced in various cell types like ovarian cells, mast cells, endometrial cells, erythrocytes, neurons, and many others [7]. LPA exerts its action via at least six high affinity, transmembrane G-protein-coupled receptor types (LPA1–6) [8]. These LPA receptors are expressed in various organs and cells.

LPA is implicated in the pathogenesis of many diseases, including atherosclerosis, neuropathic pain, and hepatic, renal, and pulmonary fibrosis. LPA activity has been associated with colorectal, breast, gastric, prostate, hepatocellular, and ovarian cancers [9]. LPA has been proposed as a potential biomarker for ovarian and other gynecological cancers because higher levels are observed in cancer patients than in healthy controls [7]. Studies in various species of animals and also humans have identified important roles for the receptor-mediated LPA signaling in multiple aspects of human and animal reproductive tract function [8]. Previous studies indicate a significant role of cytokine production (such as IL-8 and VEGF) impairment in the development of adenomyosis [1, 2].

Given the important roles of the receptor-mediated LPA signaling in both reproductive tract function and gynecological cancers, it will be informative to investigate the potential role of LPA in the development of adenomyosis. To our knowledge, no studies have investigated the role of LPA in the development of adenomyosis. In the current study, we determined LPA levels in the plasma of adenomyosis patients and healthy controls by using HPLC-ESI-MS/MS. Immunohistochemistry was performed to evaluate the expression of six LPA receptors (LPA1–6) in endometrial tissue samples collected from adenomyosis patients and healthy controls. We also assessed the effects of LPA on IL-8 production, VEGF production and cell proliferation in human endometrial stromal cells.

## Methods

### Collection of plasma samples for HPLC-ESI-MS/MS analysis of LPA

This study was approved by the Clinical Research Ethics Committee of the Jiangxi Provincial Maternal and Child Health Hospital (Registration number: JXFBEC005–2014, registered on 20th of july 2014). All subjects were given consent forms and the study protocols, and they provided written, informed consent. Plasma samples during the proliferative phase were obtained from thirty patients (age range, 30–42 years) who had been diagnosed with adenomyosis by laparoscopy and confirmed by pathological examination. Another thirty age matched fertile women (proliferative phase) who were selected from the health examination center were regarded as the control group. The sampling conditions between adenomyosis patients and healthy controls are identical. These control women were healthy (with neither clinical symptoms of adenomyosis nor any abnormality detected during the clinical examination) according to physical examination, transvaginal ultrasound, and biochemical blood tests. Women with irregular cycling, amenorrheic postmenopausal women and those who had received steroid hormone therapy in the last six months were excluded from the study.

### Tissue sections for immunohistochemistry

After informed consent was obtained, eutopic endometrial tissue and ectopic endometrial tissue (Endometrial samples were placed in 4% paraformaldehyde before paraffin embedding. Paraffin blocks were sectioned, representative slides stained with hematoxy and eosin. Ectopic endometrial tissue, with subsequent histological verification of endometriotic lesions in myometrium.) samples were obtained during the proliferative phase of the menstrual cycle from ten patients (age range, 30–42 years) with both laparoscopically and histologically diagnosed adenomyosis. Healthy endometrial tissues were collected during the proliferative phase of the menstrual cycle from ten women (age range, 30–42 years) who underwent surgery for benign indications other than adenomyosis or endometriosis. Both these ten control patients and adenomyosis subjects exhibited normal hormonal profiles.

None of the patients had received steroid hormone therapy within the past six months, and none exhibited any obvious internal medicine or surgical comorbidities. Women with irregular cycling, endometrial abnormalities, fibroids, ovarian cysts, or lesions were excluded. The fertility status of the patients used in this study was unknown.

### Collection and preparation of tissue specimens for endometrial stromal cell cultures

After informed consent was obtained, endometrial tissues were collected during hysterectomy from five women (proliferative phase, age range, 30–42 years) who had been diagnosed with intramural leiomyoma, with no evidence of endometrial abnormalities, adenomyosis, or endometriosis, and who had not taken any hormonal medication in the past six months. The endometrial tissues were placed in Hank’s balanced salt solution (HBSS) and transported to the laboratory for endometrial stromal cell (ESC) isolation and culture.

### Blood processing, LPA extraction, and HPLC-ESI-MS/MS analysis of LPA

Blood samples were collected in EDTA-containing tubes and centrifuged at 1750×g for 15 min at room temperature. Plasma samples were frozen and stored at −80 °C until used. LPA were extracted as described previously [10]. HPLC-ESI-MS/MSanalysis of LPA species were performed using a hybrid triple quadrupole/linear ion trap mass spectrometer (AB SCIEX Qtrap, Framingham, MA, USA) and interfaced with a Shimadzu HPLC system (Shimadzu, Tokyo, Japan). LPA species were separated with a reversed phase C18 column (20 mm × 2.1 mm, 5 μm, Shiseido, Japan). 300 μM ammonium phosphate buffer, pH 5.4 solution was used for mobile phase A, while 9:1 (*V*/V) methanol-acetonitrile was used as mobile phase B. The gradient was 70% B (30% A) at 0.2 mL/min, and 8 min for each sample. A [^13^C_16_] labeled 16:0 LPA is used as the internal standard. Mass spectrometric analyses were performed online using electrospray ionization tandem mass spectrometry in the multiple reaction monitoring (MRM) mode. Monitoring ions were m/z 425 → 153 for [^13^C_16_] 16:0 LPA, m/z 481 → 153 for 22:6 LPA, m/z 457 → 153 for 22:4 LPA, m/z 437 → 153 for 18:0 LPA, m/z 435 → 153 for 18:1 LPA, m/z 433 → 153 for 18:2LPA, m/z 409 → 153 for 16:0 LPA. All standard LPA forms and heavy isotope-labeled [^13^C_16_] 16:0 LPA were purchased from Avanti Polar Lipids (Alabaster, AL, USA). Quantitative analysis was performed as described previously. We established standard curves for each LPA form by mixing different concentrations of a particular form of LPA with the same concentration of internal standard, and then performing ESI-MS analyses. Data processing was highly automated by using the MS software.

### Immunohistochemistry

Representative paraffin-embedded soft tissues were cut into 4-μm-thick sections. Tissue sections were deparaffinized in xylene solutions at room temperature, hydrated in serial diluted alcohols. Antigen retrieval was then performed in citrate buffer (pH 6.0, 15 min), and endogenous peroxidase activity was eliminated by incubation in 3% hydrogen peroxide. Slides were then incubated with an anti-LPA1 antibody (LS-B1498/63066; Lifespan Bioscience Inc., Seattle, WA, 1:500 dilution, rabbit), an anti-LPA2 antibody (LS-B512/10150; Lifespan Bioscience Inc., Seattle, WA, 1:300 dilution, rabbit), an anti-LPA3 antibody (LS-A1014/40949; Lifespan Bioscience Inc., Seattle, WA, 1:50 dilution, rabbit), an anti-LPA4 antibody (LS-A872/36703; Lifespan Bioscience Inc., Seattle, WA, 1:100 dilution, rabbit), an anti-LPA5 antibody (LS-A428/42622; Lifespan Bioscience Inc., Seattle, WA, 1:50 dilution, rabbit), an anti-LPA6 antibody (LS-A852/52093; Lifespan Bioscience Inc., Seattle, WA, 1:50 dilution, rabbit) overnight at 4 °C. The primary antibody was replaced with phosphate-buffered saline (PBS) for negative control slides. On the following day, the slides were incubated with a biotin-labeled goat anti-rabbit secondary antibody (Zhongshanjinqiao Biotec, Beijing, China). To visualized protein expression, a chromogenic reaction was performed using a 3,3′-diaminobenzidine color reagent kit (ZSGB-BIO, Beijing, China) according to the manufacturer’s instructions. Image-Pro plus software was used to quantitative analyze the results as described previously [11]. With reference to Weidner’s counting method, any endothelial cell or endothelial cell cluster that was clearly separate from an adjacent cluster was considered to be a single, countable microvessel. The microvessel density (MVD) values are expressed as the largest number of microvessels.

### Isolation and culture of human ESCs

Endometrial tissue was minced with a sterile surgical blade and digested in HBSS (Sigma-Aldrich, St. Louis, USA) containing collagenase B (1 mg/mL, 15 IU/mg), deoxyribonuclease I (0.1 mg/mL, 1500 IU/mg), penicillin (200 U/mL), and streptomycin (200 mg/mL) for 1 h at 37 °C with gentle shaking. The dispersed endometrial cells were separated by filtration through a 75-μm wire sieve, and the resultant supernatant was centrifuged at 1000 rmp for 5 min, the pellets (ESCs) was resuspended and cultured in a solution of Dulbecco’s modified Eagle’s medium (DMEM) and Ham’s F-12 (1:1 *v*/v; Invitrogen Corp. Shanghai, China), containing fetal bovine serum (10% v/v; ExCell Biology Inc. Shanghai, China). The cultures were maintained under a standard 95% atmosphere, in a 5% CO_2_ incubator at 37 °C, and allowed to replicate to confluence. ESCs were cultured in DMEM supplemented with 10% fetal bovine serum in a humidified atmosphere of 95% air-5% CO_2_ at 37 °C. After 1 passage, ESCs were assayed immunocytochemically, using vimentin and cytokeratin as specific cell-surface markers, and confirmed to have a purity of >95%.

### Assay of proliferation of ESCs

Cell proliferation was determined by using a cell count-8 kit (Dojindo Lab. Tokyo, Japan) based on the protocol described previously [12]. ESCs were seeded at 1× 10^4^ per well in 200 μL medium. Cells were exposed to 0.1, 1, 10 μM LPA for 24 h or 48 h. WST-8 reagent solution (10 μL) was added to each well, and then the mixture was incubated for 2 h at 37 °C under an atmosphere of 5% CO_2_. Absorbance of formazan in cell suspension was measured at 450 nm using a microplate spectrophotometer (BioRad, CA, USA).

### IL-8 and VEGF assay by ELISA

ESCs were grown in 6-well plates (1× 10^6^ per well) in 2 ml of medium. Cells were exposed to 0.1, 1, 10 μM LPA for 24 h or 48 h. After treatment, the cell suspensions were centrifuged, and the supernatant was collected and stored at −80 °C until analysis. IL-8 and VEGF levels in cell-free supernatants were measured using commercially available ELISA kit (R&DSystems, Minneapolis, MN, USA) according to manufacturer’s instructions.

### Statistical analysis

The results of measurement were expressed as mean ± standard deviation (SD). Statistical analysis were performed with one-way analysis of variance followed by Tukey’s post hoc test or an independent samples t test. All statistical analyses were conducted with the SPSS 20 statistical software package. *P* values of <0.05 were considered significant.

## Results

### Plasma LPA levels is significantly increased in patients with Adenomyosis

Using HPLC-ESI-MS/MS method, we quantify the levels of six LPA species (16:0, 18:2, 18:1, 18:0, 20:4 and 22:6 LPA) in plasma samples collected from adenomyosis patients and healthy controls. As shown in Table. 1, the adenomyosis patients group showed significantly higher plasma levels of total LPA and LPA species 16:0-, 18:2-, 18:1-, 20:4-, 22:6-LPA, compared with controls. 18:2 LPAwas the predominant form in all plasma samples. We also find that the levels of LPA species in human plasma generally follow the order: 18:2 LPA > 16:0 LPA, 20:4 LPA >18:1 LPA, 22:6 LPA >18:0 LPA.

**Table 1:** Comparison of plasma LPA levels (μM) between adenomyosis patients and healthy controls

LPA forms	Adenomyosis patients (*n* = 30)	Healthy controls (n = 30)	*P*
22:6 LPA	0.129 ± 0.0352	0.0923 ± 0.0227	<0.001
20:4 LPA	0.231 ± 0.0511	0.161 ± 0.0514	<0.001
18:0 LPA	0.0228 ± 0.00974	0.0217 ± 0.00641	0.76
18:1 LPA	0.122 ± 0.0307	0.0917 ± 0.0186	0.004
18:2 LPA	1.13 ± 0.225	0.579 ± 0.166	<0.001
16:0 LPA	0.402 ± 0.0748	0.309 ± 0.0998	0.009
Total LPA	2.04 ± 0.427	1.26 ± 0.365	<0.001

### Aberrant expression of LPA receptors in the endometria of patients with Adenomyosis

**Fig. 1:**
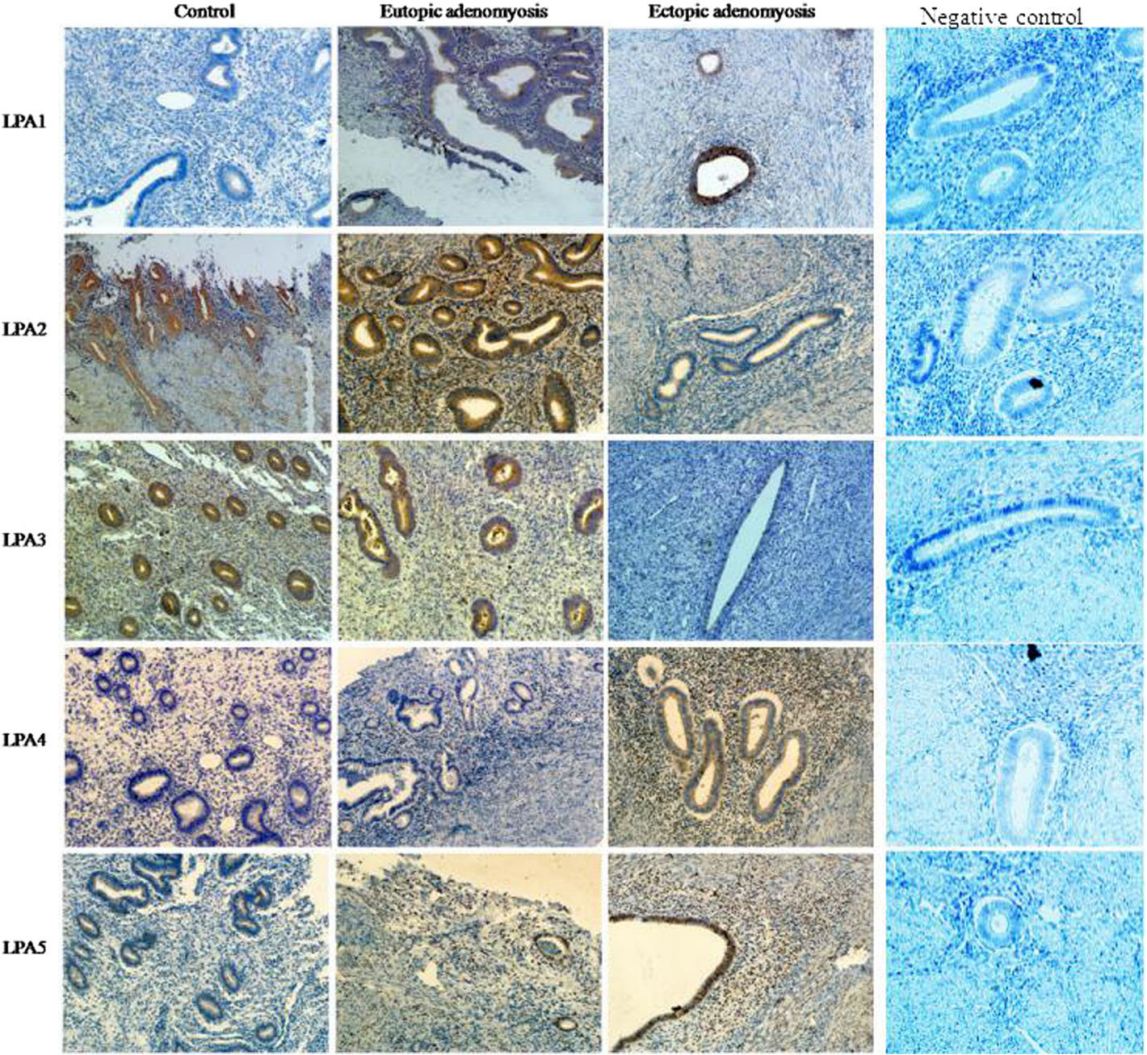
Representative micrographs (×400) of five LPA receptors (LPA1–5) immunostaining in the endometria of control subjects (n = 10) or in the eutopic and ectopic endometria of patients with adenomyosis (*n* = 10)

**Fig. 2:**
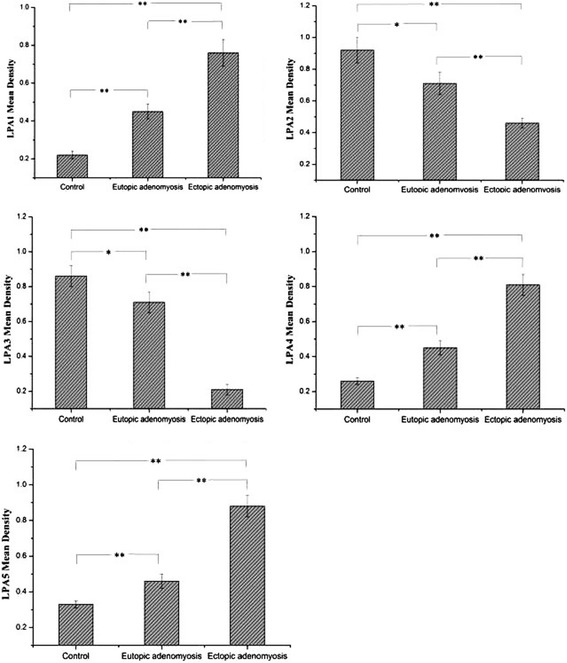
Summary of five LPA receptors (LPA1–5) expression and statistical comparison of five LPA receptors (LPA1–5) expression in the endometrium from control subjects (n = 10) and patients with adenomyosis (n = 10). *P < 0.05, **P < 0.01

To investigate the potential role of LPA receptors in adenomyosis, we used IHC to examine the expression of the LPA receptors LPA1–6 in the ectopic endometrium and paired eutopic endometrium in patients with adenomyosis and in the endometrial of healthy controls. Herein, we show endometrial express LPA receptors LPA1–5, while LPA6 was undetectable in the endometria of both patients with adenomyosis and healthy controls (data of LPA6 not shown).As shown in Fig. 1, In endometrium in healthy control group, LPA1, LPA4 and LPA5 staining were little in both stroma and epithelial cells, LPA2 and LPA3 staining were localized to the nuclei of both stromal and glandular cells. In eutopic endometrium in patients with adenomyosis group, LPA1 staining was localized to the cytoplasm, membrances of the epithelial cells of the endometrial glands, LPA2, LPA3 and LPA5 were localized to the nuclei of both stromal and glandular cells, LPA4 was little in both stroma and epithelial cells. In ectopic endometrium in patients with adenomyosis group, LPA1 staining was localized to the cytoplasm, membrances of the epithelial cells of the endometrial glands, LPA2, LPA4 and LPA5 staining were localized to the nuclei of both stromal and glandular cells, LPA3 was little in both stroma and epithelial cells. As shown in Fig. 1 and Fig. 2, LPA1, LPA4 and LPA5 immunoreactivity were significantly higher in the adenomyosis group than in the control group, while LPA2 and LPA3 immunoreactivity were significantly lower in the adenomyosis group than in the control group. In addition, the levels of LPA1, LPA4 and LPA5 in the ectopic endometrium were much higher than in the eutopic endometrium in the adenomyosis group, while the levels of LPA2 and LPA3 in the ectopic endometrium were much lower than in the eutopic endometrium in the adenomyosis group.

### LPA induces the release of IL-8 from ESCs

**Fig. 3:**
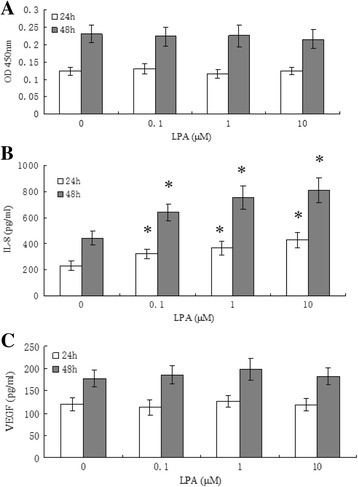
The effects of LPA on cell proliferation (**a**), IL-8 production (**b**) and VEGF production (**c**) in human endometrial stromal cells. Data represent mean ± SD from 4 independent experiments, each consisting of 2 samples (*n* = 5 × 2). *P < 0.01, indicating statistically significant difference between LPA-treated groups and control group

As shown in Fig. 3, LPA increase the release of IL-8 from ESCs, while no differences in the release of VEGF from ESCs were detected in LPA treatment group compared with control. LPA at all concentrations did not affect cell proliferation after 24 h or 48 h stimulation.

## Discussion

This study is the first to show elevated plasma levels of LPA and aberrant expression of LPA receptors in adenomyosis. In addition, LPA significantly induced the release of IL-8 from ESCs but did not affect cell proliferation and VEGF production. These findings presented in this study provide novel insight into the molecular mechanisms and biomarkers of adenomyosis.

LPA present in the plasma of healthy women at a range of 0.1–6.3 μM as reported by Xu Y et al. [13]. LPA levels were found to be elevated in the plasma of more than 90% of ovarian cancer patients [14]. Its levels were also found elevated in the plasma of patients with cervical and endometrial cancer [15],as well as multiple myeloma [16]. We report here that plasma levels of different LPA species are significant increased in adenomyosis patients compared with unaffected controls, suggesting that the increase in plasma LPA seen in adenomyosis patients may reflect pathophysiological changes in adenomyosis development.

LPA exerts its action via at least six high affinity, transmembrane G-protein-coupled receptor types (LPA1–6). As shown in Fig. 1 and Fig. 2, LPA1, LPA4 and LPA5 immunoreactivity were significantly higher in the adenomyosis group than in the control group, while LPA2 and LPA3 immunoreactivity were significantly lower in the adenomyosis group than in the control group.

Women with adenomyosis have a significantly decreased clinical pregnancy rate and a significantly increased miscarriage rate, contributing to the significant decrease in the final live birth rate, and suggest that there is a strong association between adenomyosis and subfertility [17, 18]. Studies in various species of animals and also in humans have identified important roles for the receptor-mediated LPA signaling in multiple aspects of human and animal reproductive tract function [8].These aspects range from ovarian and uterine function, estrous cycle regulation, early embryo development, embryo implantation, decidualization to pregnancy maintenance and parturition. By extrapolation, elevated plasma levels of LPA and aberrant expression of LPA receptors may be one important factor leading to infertility in women with adenomyosis.

Adenomyosis is characterized by the presence of ectopic endometrial tissue, including glands and stroma, within the myometrium. It is well accepted that adenomyosis exhibits features similar to that of tumor metastasis, as it is defined by progressive invasion by the endometrium and by the formation of ectopic lesions. Though a benign condition, clinical and microscopic adenomyosis appears to act like cancer characterized by cell invasion, uncontrolled growth, neovascularization.^1^ In both in vivo and in vitro systems, LPA has been shown to participate in each of these processes, underscoring the involvement LPA and the potential therapeutic benefit of targeting LPA signaling. LPA signaling are implicated in the cancer field as important factors in the migration, invasion, metastasis, proliferation, survival, and angiogenesis processes of tumorigenic cells [19]. Our study suggesting that elevated plasma levels of LPA and alteration of LPA receptor expression might be an important event in the development of adenomyosis.

The pain syndrome represents the major clinical problem of adenomyosis. There remains no commonly accepted mechanism to explain how adenomyosis might cause pain. LPA has been shown to participate in pain generation [20]. In mouse models, LPA has been shown elicit pain when administrated locally to the hind paw. The relation between LPA and pain caused by adenomyosis merits further investigation.

Ectopic endometrial tissue is involved in the pathogenesis of adenomyosis [21]. It has been reported that apoptosis is reduced in the ectopic endometrium compared with the eutopic endometrium in adenomyosis [22]. It has been reported that the hydroxyl radical released by the ectopic endometrium is intimately involved in the pathology of adenomyosis. Ectopic mononuclear cells of women with adenomyosis produced higher levels of IFNγ, IFNα and TNFα than the mononuclear cells of eutopic endometrium [23]. In the present study, the levels of LPA1, LPA4 and LPA5 in the ectopic endometrium were much higher than in the eutopic endometrium in the adenomyosis group, while the levels of LPA2 and LPA3 in the ectopic endometrium were much lower than in the eutopic endometrium in the adenomyosis group. These results indicate that the differently expressed LPA receptors between eutopic and ectopic endometrium in the adenomyosis might be an important event in the development of adenomyosis.

We also assessed the effects of LPA on cell proliferation, IL-8 and VEGF production in ESCs. We found that LPA significantly enhances the release of IL-8 from ESCs but does not affect cell proliferation and VEGF production. IL-8, known as an α-chemokine that shows neutrophil chemotactic/activating and T-cell chemotactic activity both in vivo and in vitro, is believed to be significant in endometrial physiology and adenomyosis. It has been shown that, in addition to the immune cells, endometrium is also one of the sources of the IL-8. Previous study revealed that epithelial cells of adenomyosis foci express significantly higher levels IL-8 and MCP-1 compared with their corresponding eutopic endometrium [24]. Ulukus et al. [25] reported higher epithelial IL-8 and MCP-1 in eutopic endometrium in endometriosis compared with control endometrium. It has been shown that both IL-8 and MCP-1 levels are increased in the peritoneal fluid of women with endometriosis [26]. It has also been demonstrated that IL-8 significantly stimulates cell proliferation in endometrial stromal cells. Our results showing higher IL-8 production in ESCs exposed to LPA support potential roles of LPA in pathogenesis of adenomyosis. However, all the experiments were performed with ESCs, and it is unclear how those cells compare to human endometrial epithelial cells or in vivo situations. Further investigation is clearly warranted to examine whether LPA can cause such effects in human endometrial epithelial cells or in vivo situations.

## Conclusion

In summary, this study is the first to show elevated plasma levels of LPA and aberrant expression of LPA receptors in adenomyosis. In addition, LPA significantly induced the release of IL-8 from ESCs. These results indicate that the receptor-mediated LPA signaling may play an important role in the development of adenomyosis.

## Abbreviations

ESCs: human endometrial stromal cells
HPLC-ESI-MS/MS: high-performance liquid chromatography electrospray ionization tandem mass spectrometry
IL-8: interleukin-8
LPA: lysophosphatidic acid
LPA1–6: six LPA receptors
VEGF: vascular endothelial growth factor
